# *cis*-(Di-2-pyridyl­amine-κ^2^ *N* ^2^,*N* ^2′^)bis­(thio­cyanato-κ*S*)platinum(II)

**DOI:** 10.1107/S1600536812010926

**Published:** 2012-03-17

**Authors:** Kwang Ha

**Affiliations:** a School of Applied Chemical Engineering, The Research Institute of Catalysis, Chonnam National University, Gwangju 500-757, Republic of Korea

## Abstract

In the title complex, [Pt(NCS)_2_(C_10_H_9_N_3_)], the Pt^II^ ion is four-coordinated in a distorted square-planar environment by the two pyridine N atoms of the chelating di-2-pyridyl­amine (dpa) ligand and two mutually *cis* S atoms from two linear thio­cyanate anions. The dpa ligand is not planar, the dihedral angle between the pyridine rings being 30.8 (4)°. In the crystal, the complex mol­ecules are stacked in columns along the *a* axis and are connected by inter­molecular N—H⋯N hydrogen bonds, forming supra­molecular chains along the *b* axis.

## Related literature

For the crystal structure of the related chlorido Pt^II^ complex [PtCl_2_(dpa)], see: Li & Liu (2004 ▶); Tu *et al.* (2004 ▶); Zhang *et al.* (2006 ▶).
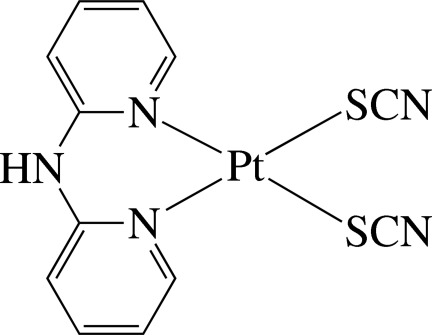


## Experimental

### 

#### Crystal data

[Pt(NCS)_2_(C_10_H_9_N_3_)]
*M* *_r_* = 482.45
Triclinic, 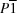

*a* = 7.2282 (6) Å
*b* = 9.8308 (8) Å
*c* = 10.2501 (8) Å
α = 94.292 (2)°
β = 93.081 (2)°
γ = 106.123 (2)°
*V* = 695.64 (10) Å^3^
*Z* = 2
Mo *K*α radiation
μ = 10.38 mm^−1^
*T* = 200 K
0.19 × 0.15 × 0.09 mm

#### Data collection

Bruker SMART 1000 CCD diffractometer
Absorption correction: multi-scan (*SADABS*; Bruker, 2000 ▶) *T*_min_ = 0.812, *T*_max_ = 1.000
4195 measured reflections
2636 independent reflections
2391 reflections with *I* > 2σ(*I*)
*R*_int_ = 0.018

#### Refinement

*R*[*F* ^2^ > 2σ(*F* ^2^)] = 0.031
*wR*(*F* ^2^) = 0.088
*S* = 1.22
2636 reflections
181 parameters
H-atom parameters constrained
Δρ_max_ = 3.96 e Å^−3^
Δρ_min_ = −1.40 e Å^−3^

### 

Data collection: *SMART* (Bruker, 2000 ▶); cell refinement: *SAINT* (Bruker, 2000 ▶); data reduction: *SAINT*; program(s) used to solve structure: *SHELXS97* (Sheldrick, 2008 ▶); program(s) used to refine structure: *SHELXL97* (Sheldrick, 2008 ▶); molecular graphics: *ORTEP-3* (Farrugia, 1997 ▶) and *PLATON* (Spek, 2009 ▶); software used to prepare material for publication: *SHELXL97*.

## Supplementary Material

Crystal structure: contains datablock(s) global, I. DOI: 10.1107/S1600536812010926/tk5067sup1.cif

Structure factors: contains datablock(s) I. DOI: 10.1107/S1600536812010926/tk5067Isup2.hkl

Additional supplementary materials:  crystallographic information; 3D view; checkCIF report

## Figures and Tables

**Table:** 

Pt1—N1	2.065 (7)
Pt1—N3	2.069 (7)
Pt1—S2	2.302 (2)
Pt1—S1	2.306 (2)

**Table:** 

N1—Pt1—N3	88.1 (3)
S2—Pt1—S1	89.04 (9)

**Table 2:** Hydrogen-bond geometry (Å, °)

*D*—H⋯*A*	*D*—H	H⋯*A*	*D*⋯*A*	*D*—H⋯*A*
N2—H2*N*⋯N5^i^	0.92	1.93	2.851 (11)	176

Symmetry code: (i) 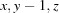.

**Fig. 1.**
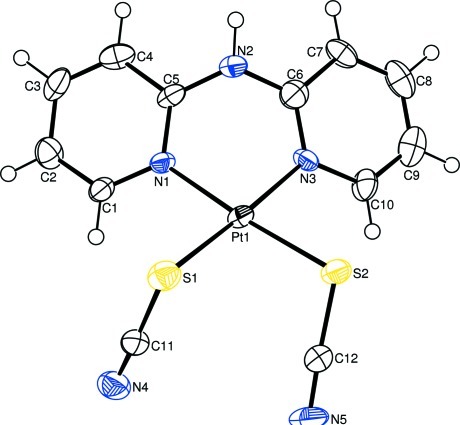
The molecular structure of the title complex, with displacement ellipsoids drawn at the 50% probability level for non-H atoms.

**Fig. 2.**
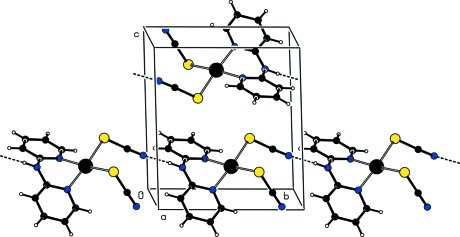
A view of the unit-cell contents of the title complex. Intermolecular N—H···N hydrogen-bond interactions are drawn with dashed lines.

## supplementary crystallographic information

### Comment

Crystal structures of the related chlorido Pt^II^ complex, [PtCl_2_(dpa)] (dpa = di-2-pyridylamine, C_10_H_9_N_3_), have been reported previously (Li & Liu, 2004; Tu *et al.*, 2004; Zhang *et al.*, 2006).

In the title complex, [Pt(NCS)_2_(dpa)], the Pt^II^ ion is four-coordinated in a distorted square-planar environment by the two pyridyl N atoms of the chelating dpa ligand and two S atoms from two thiocyanate anions (Fig. 1). The dpa ligand is not planar with the dihedral angle between the least-squares planes of the pyridyl rings being 30.8 (4)°. The thiocyanato ligands are located on the same sides of the PtS_2_N_2_ plane and are almost linear with the bond angles <S1—C11—N4 = 177.4 (9)° and <S2—C12—N5 = 177.3 (9)°. The pairs of Pt—N and Pt—S bond lengths are nearly equivalent (Table 1). The complex molecules are stacked in columns along the *a* axis and are connected by intermolecular N—H···N hydrogen bonds, forming chains along the *b* axis (Fig. 2 and Table 2). In the columns, intermolecular π-π interactions between the pyridine rings are present, the ring centroid-centroid distance being 4.155 (5) Å.

### Experimental

To a solution of K_2_PtCl_4_ (0.2066 g, 0.498 mmol) in H_2_O (20 ml) and MeOH (10 ml) were added KSCN (0.5232 g, 5.384 mmol) and di-2-pyridylamine (0.0883 g, 0.516 mmol) and stirred for 7 h at room temperature. The formed precipitate was separated by filtration and washed with H_2_O and MeOH, and dried at 50 °C, to give a yellow powder (0.2182 g). Crystals suitable for X-ray analysis were obtained by slow evaporation from a CH_3_CN solution.

### Refinement

Carbon-bound H atoms were positioned geometrically and allowed to ride on their respective parent atoms [C—H = 0.95 Å and *U*_iso_(H) = 1.2*U*_eq_(C)]. Nitrogen-bound H atom was located from Fourier difference maps then allowed to ride on its parent atom in the final cycles of refinement with N—H = 0.92 Å and *U*_iso_(H) = 1.5 *U*_eq_(N). The highest peak (3.96 e Å^-3^) and the deepest hole (-1.40 e Å^-3^) in the difference Fourier map are located 0.98 Å and 0.99 Å from the Pt1 atom, respectively. Owing to poor agreement, the following reflections were omitted from the final refinement: (8 7 2), (0 11 4), (7 8 4), (2 99), (70 8), (7 5 7), (8 7 3), (8 8 0), (0 9 8), (7 4 7), (6 8 6), (8 6 3), (5 7 8), (4 7 9), (3 1 11), ( 66 2), (8 8 1), (8 7 1), (1 9 9), (0 10 5), (7 7 5), (2 4 11), (8\14), (48 8), (8 7 2), (2 5 11), (3 2 11), (1 10 4), and (2 11 4).

### Crystal data

**Table:**

[Pt(NCS)_2_(C_10_H_9_N_3_)]	*Z* = 2
*M**_r_* = 482.45	*F*(000) = 452
Triclinic, *P*1	*D*_x_ = 2.303 Mg m^−^^3^
Hall symbol: -P 1	Mo *K*α radiation, λ = 0.71073 Å
*a* = 7.2282 (6) Å	Cell parameters from 3201 reflections
*b* = 9.8308 (8) Å	θ = 2.8–25.9°
*c* = 10.2501 (8) Å	µ = 10.38 mm^−^^1^
α = 94.292 (2)°	*T* = 200 K
β = 93.081 (2)°	Block, yellow
γ = 106.123 (2)°	0.19 × 0.15 × 0.09 mm
*V* = 695.64 (10) Å^3^	

### Data collection

**Table:**

Bruker SMART 1000 CCD diffractometer	2636 independent reflections
Radiation source: fine-focus sealed tube	2391 reflections with *I* > 2σ(*I*)
Graphite monochromator	*R*_int_ = 0.018
φ and ω scans	θ_max_ = 26.0°, θ_min_ = 2.0°
Absorption correction: multi-scan (*SADABS*; Bruker, 2000)	*h* = −8→8
*T*_min_ = 0.812, *T*_max_ = 1.000	*k* = −12→11
4195 measured reflections	*l* = −12→12

### Refinement

**Table:**

Refinement on *F*^2^	Primary atom site location: structure-invariant direct methods
Least-squares matrix: full	Secondary atom site location: difference Fourier map
*R*[*F*^2^ > 2σ(*F*^2^)] = 0.031	Hydrogen site location: inferred from neighbouring sites
*wR*(*F*^2^) = 0.088	H-atom parameters constrained
*S* = 1.22	*w* = 1/[σ^2^(*F*_o_^2^) + (0.0179*P*)^2^ + 8.2228*P*] where *P* = (*F*_o_^2^ + 2*F*_c_^2^)/3
2636 reflections	(Δ/σ)_max_ = 0.001
181 parameters	Δρ_max_ = 3.96 e Å^−^^3^
0 restraints	Δρ_min_ = −1.40 e Å^−^^3^

### Special details

**Table:**

Geometry. All e.s.d.'s (except the e.s.d. in the dihedral angle between two l.s. planes) are estimated using the full covariance matrix. The cell e.s.d.'s are taken into account individually in the estimation of e.s.d.'s in distances, angles and torsion angles; correlations between e.s.d.'s in cell parameters are only used when they are defined by crystal symmetry. An approximate (isotropic) treatment of cell e.s.d.'s is used for estimating e.s.d.'s involving l.s. planes.
Refinement. Refinement of *F*^2^ against ALL reflections. The weighted *R*-factor *wR* and goodness of fit *S* are based on *F*^2^, conventional *R*-factors *R* are based on *F*, with *F* set to zero for negative *F*^2^. The threshold expression of *F*^2^ > σ(*F*^2^) is used only for calculating *R*-factors(gt) *etc*. and is not relevant to the choice of reflections for refinement. *R*-factors based on *F*^2^ are statistically about twice as large as those based on *F*, and *R*- factors based on ALL data will be even larger.

### Fractional atomic coordinates and isotropic or equivalent isotropic displacement parameters (Å^2^)

**Table:**

	*x*	*y*	*z*	*U*_iso_*/*U*_eq_	
Pt1	1.01671 (5)	0.50573 (3)	0.26893 (3)	0.02699 (12)	
S1	1.3097 (3)	0.6767 (2)	0.2720 (2)	0.0351 (5)	
S2	0.9284 (4)	0.6445 (2)	0.4314 (2)	0.0367 (5)	
N1	1.0971 (10)	0.3699 (7)	0.1344 (7)	0.0256 (15)	
N2	0.9426 (11)	0.1761 (8)	0.2524 (7)	0.0307 (16)	
H2N	0.9464	0.0901	0.2805	0.046*	
N3	0.7667 (10)	0.3445 (7)	0.2845 (7)	0.0254 (15)	
N4	1.2719 (12)	0.8295 (9)	0.0535 (9)	0.043 (2)	
N5	0.9605 (17)	0.9069 (9)	0.3291 (10)	0.060 (3)	
C1	1.2059 (12)	0.4162 (9)	0.0350 (9)	0.0293 (19)	
H1	1.2247	0.5119	0.0157	0.035*	
C2	1.2890 (14)	0.3335 (10)	−0.0382 (9)	0.036 (2)	
H2	1.3633	0.3698	−0.1081	0.044*	
C3	1.2635 (14)	0.1923 (11)	−0.0089 (10)	0.041 (2)	
H3	1.3234	0.1322	−0.0570	0.050*	
C4	1.1520 (13)	0.1437 (9)	0.0890 (9)	0.034 (2)	
H4	1.1351	0.0490	0.1107	0.041*	
C5	1.0619 (12)	0.2315 (9)	0.1582 (9)	0.0272 (18)	
C6	0.7784 (14)	0.2105 (10)	0.2872 (9)	0.034 (2)	
C7	0.6286 (13)	0.1040 (10)	0.3285 (10)	0.038 (2)	
H7	0.6401	0.0104	0.3319	0.045*	
C8	0.4652 (15)	0.1353 (12)	0.3638 (10)	0.048 (3)	
H8	0.3619	0.0636	0.3925	0.058*	
C9	0.4500 (14)	0.2743 (12)	0.3578 (9)	0.042 (2)	
H9	0.3369	0.2980	0.3818	0.051*	
C10	0.6011 (12)	0.3736 (11)	0.3166 (9)	0.035 (2)	
H10	0.5906	0.4670	0.3101	0.042*	
C11	1.2825 (13)	0.7673 (10)	0.1414 (10)	0.035 (2)	
C12	0.9486 (13)	0.7994 (10)	0.3678 (9)	0.033 (2)	

### Atomic displacement parameters (Å^2^)

**Table:**

	*U*^11^	*U*^22^	*U*^33^	*U*^12^	*U*^13^	*U*^23^
Pt1	0.0312 (2)	0.02336 (19)	0.0312 (2)	0.01455 (14)	0.00534 (13)	0.00443 (13)
S1	0.0337 (12)	0.0298 (12)	0.0411 (13)	0.0094 (10)	−0.0007 (10)	−0.0008 (10)
S2	0.0573 (15)	0.0285 (12)	0.0319 (12)	0.0220 (11)	0.0132 (11)	0.0064 (9)
N1	0.025 (4)	0.017 (3)	0.036 (4)	0.007 (3)	0.005 (3)	0.003 (3)
N2	0.039 (4)	0.023 (4)	0.034 (4)	0.014 (3)	0.006 (3)	0.006 (3)
N3	0.025 (4)	0.021 (3)	0.029 (4)	0.003 (3)	0.004 (3)	0.009 (3)
N4	0.031 (4)	0.033 (5)	0.064 (6)	0.006 (4)	0.005 (4)	0.014 (4)
N5	0.100 (8)	0.028 (5)	0.066 (7)	0.033 (5)	0.030 (6)	0.018 (4)
C1	0.027 (4)	0.025 (4)	0.038 (5)	0.012 (4)	0.005 (4)	0.002 (4)
C2	0.041 (5)	0.037 (5)	0.028 (5)	0.007 (4)	0.007 (4)	−0.002 (4)
C3	0.030 (5)	0.046 (6)	0.053 (6)	0.020 (5)	0.007 (5)	−0.002 (5)
C4	0.039 (5)	0.021 (4)	0.039 (5)	0.007 (4)	−0.012 (4)	−0.007 (4)
C5	0.025 (4)	0.021 (4)	0.037 (5)	0.010 (3)	−0.003 (4)	−0.003 (3)
C6	0.039 (5)	0.039 (5)	0.028 (5)	0.015 (4)	0.001 (4)	0.003 (4)
C7	0.033 (5)	0.028 (5)	0.044 (6)	−0.008 (4)	0.004 (4)	0.010 (4)
C8	0.035 (6)	0.056 (7)	0.042 (6)	−0.007 (5)	0.007 (5)	0.010 (5)
C9	0.032 (5)	0.068 (7)	0.025 (5)	0.018 (5)	−0.006 (4)	−0.011 (5)
C10	0.022 (4)	0.047 (6)	0.040 (5)	0.014 (4)	0.004 (4)	0.005 (4)
C11	0.031 (5)	0.024 (5)	0.050 (6)	0.008 (4)	0.011 (4)	0.006 (4)
C12	0.036 (5)	0.027 (5)	0.038 (5)	0.012 (4)	0.009 (4)	0.003 (4)

### Geometric parameters (Å, º)

**Table:**

Pt1—N1	2.065 (7)	C1—H1	0.9500
Pt1—N3	2.069 (7)	C2—C3	1.406 (14)
Pt1—S2	2.302 (2)	C2—H2	0.9500
Pt1—S1	2.306 (2)	C3—C4	1.352 (14)
S1—C11	1.694 (10)	C3—H3	0.9500
S2—C12	1.674 (9)	C4—C5	1.394 (12)
N1—C1	1.349 (11)	C4—H4	0.9500
N1—C5	1.356 (10)	C6—C7	1.390 (13)
N2—C5	1.371 (11)	C7—C8	1.361 (15)
N2—C6	1.379 (12)	C7—H7	0.9500
N2—H2N	0.9200	C8—C9	1.407 (16)
N3—C6	1.346 (11)	C8—H8	0.9500
N3—C10	1.356 (11)	C9—C10	1.358 (14)
N4—C11	1.137 (12)	C9—H9	0.9500
N5—C12	1.139 (12)	C10—H10	0.9500
C1—C2	1.347 (12)		
			
N1—Pt1—N3	88.1 (3)	C2—C3—H3	120.6
N1—Pt1—S2	175.3 (2)	C3—C4—C5	120.5 (9)
N3—Pt1—S2	89.9 (2)	C3—C4—H4	119.7
N1—Pt1—S1	92.6 (2)	C5—C4—H4	119.7
N3—Pt1—S1	173.6 (2)	N1—C5—N2	121.2 (7)
S2—Pt1—S1	89.04 (9)	N1—C5—C4	120.0 (8)
C11—S1—Pt1	103.7 (3)	N2—C5—C4	118.7 (8)
C12—S2—Pt1	104.4 (3)	N3—C6—N2	120.6 (8)
C1—N1—C5	118.6 (7)	N3—C6—C7	121.3 (9)
C1—N1—Pt1	122.7 (5)	N2—C6—C7	118.1 (9)
C5—N1—Pt1	118.0 (6)	C8—C7—C6	119.3 (10)
C5—N2—C6	127.6 (7)	C8—C7—H7	120.3
C5—N2—H2N	118.5	C6—C7—H7	120.3
C6—N2—H2N	111.5	C7—C8—C9	119.7 (9)
C6—N3—C10	118.9 (8)	C7—C8—H8	120.2
C6—N3—Pt1	118.7 (6)	C9—C8—H8	120.2
C10—N3—Pt1	121.3 (6)	C10—C9—C8	118.2 (9)
C2—C1—N1	123.3 (8)	C10—C9—H9	120.9
C2—C1—H1	118.4	C8—C9—H9	120.9
N1—C1—H1	118.4	N3—C10—C9	122.6 (9)
C1—C2—C3	118.5 (9)	N3—C10—H10	118.7
C1—C2—H2	120.7	C9—C10—H10	118.7
C3—C2—H2	120.7	N4—C11—S1	177.4 (9)
C4—C3—C2	118.8 (9)	N5—C12—S2	177.3 (9)
C4—C3—H3	120.6		
			
N1—Pt1—S1—C11	84.1 (4)	C1—N1—C5—C4	6.0 (12)
S2—Pt1—S1—C11	−100.3 (3)	Pt1—N1—C5—C4	−164.9 (6)
N3—Pt1—S2—C12	−129.1 (4)	C6—N2—C5—N1	35.0 (13)
S1—Pt1—S2—C12	57.3 (4)	C6—N2—C5—C4	−146.8 (9)
N3—Pt1—N1—C1	149.1 (7)	C3—C4—C5—N1	−4.9 (13)
S1—Pt1—N1—C1	−37.3 (7)	C3—C4—C5—N2	176.8 (8)
N3—Pt1—N1—C5	−40.4 (6)	C10—N3—C6—N2	178.5 (8)
S1—Pt1—N1—C5	133.2 (6)	Pt1—N3—C6—N2	−13.8 (11)
N1—Pt1—N3—C6	40.9 (7)	C10—N3—C6—C7	−3.3 (13)
S2—Pt1—N3—C6	−134.9 (6)	Pt1—N3—C6—C7	164.4 (7)
N1—Pt1—N3—C10	−151.7 (7)	C5—N2—C6—N3	−34.6 (14)
S2—Pt1—N3—C10	32.5 (7)	C5—N2—C6—C7	147.1 (9)
C5—N1—C1—C2	−3.2 (13)	N3—C6—C7—C8	1.5 (15)
Pt1—N1—C1—C2	167.3 (7)	N2—C6—C7—C8	179.8 (9)
N1—C1—C2—C3	−0.8 (14)	C6—C7—C8—C9	0.2 (15)
C1—C2—C3—C4	1.9 (14)	C7—C8—C9—C10	−0.2 (15)
C2—C3—C4—C5	0.9 (14)	C6—N3—C10—C9	3.4 (13)
C1—N1—C5—N2	−175.9 (8)	Pt1—N3—C10—C9	−164.0 (7)
Pt1—N1—C5—N2	13.2 (10)	C8—C9—C10—N3	−1.6 (14)

### Hydrogen-bond geometry (Å, º)

**Table:**

*D*—H···*A*	*D*—H	H···*A*	*D*···*A*	*D*—H···*A*
N2—H2*N*···N5^i^	0.92	1.93	2.851 (11)	176

Symmetry code: (i) *x*, *y*−1, *z*.
